# School nurses’ perceptions and experiences of delivering a universal health-promotion program targeting both children and parents in the Swedish primary school context

**DOI:** 10.1186/s12912-025-03806-2

**Published:** 2025-09-02

**Authors:** Marianna Moberg, Åsa Norman, Liselotte Schäfer Elinder, Susanne Andermo

**Affiliations:** 1 Department of Nursing, Karolinska Institutet, Stockholm, Sweden; 2 Department of Clinical Neuroscience, Karolinska Institutet, Stockholm, Sweden; 3 Department of Women’s and Children’s Health, Uppsala University, Uppsala, Sweden; 4 Department of Global Public Health, Karolinska Institutet, Stockholm, Sweden; 5 Centre for Epidemiology and Community Medicine, Region Stockholm, Sweden; 6 Department of Physical Activity and Health, The Swedish School of Sport and Health Sciences, Stockholm, Sweden

**Keywords:** School nurse, Health promotion, Healthy school start, Parental support

## Abstract

**Background:**

Schools are important settings for universal child health promotion. Understanding the experiences of providers of interventions targeting both children and their parents is crucial for designing feasible and sustainable school-based health promotion programs.

**Aim:**

The aim of this study was to explore school nurses’ perceptions and experiences of delivering a health-promotion program focusing on healthy lifestyle habits in primary schools in Sweden.

**Methods:**

This study employed an explorative qualitative design with an inductive approach. Interviews were conducted with school nurses using a semi-structured interview guide. The data were audio-recorded, transcribed and analysed using thematic analysis.

**Results:**

School nurses had insights regarding the acceptability and appropriateness of the program as well as reflections regarding implementation and perceived impact. School nurses appreciated the evidence-based content and structure of the program and experienced improved collaboration with teachers. Moreover, positive changes were noted in relation to both children and parents such as improved knowledge and relations. Declining interest from school principals over the school year and lack of involvement among some parents were highlighted as implementation challenges.

**Conclusion:**

This study sheds light on the complexities faced by school nurses in delivering health-promotion interventions in collaboration with teachers in primary schools. Evidence-based practices, sustained leadership support and culturally sensitive strategies are essential for successful health promotion within schools. Our findings underscore the critical role of school nurses as health advocates, educators, and facilitators.

**Supplementary Information:**

The online version contains supplementary material available at 10.1186/s12912-025-03806-2.

## Background

The latest report from the Swedish Public Health Agency on school children’s health behaviours in Sweden reveals several concerning trends [1]. Specifically, in terms of food and physical activity, the report highlights that while vegetable consumption has increased it is still only half of the recommended intake, the intake of sugar is well over the recommended intake, and fewer children eat breakfast daily. Additionally, most children do not meet recommendations on physical activity, spending much of their free time sitting. Consequently, the prevalence of childhood overweight and obesity has doubled over the past three decades, with children in socioeconomically disadvantaged areas being more likely to be affected by obesity than those in more affluent areas [2]. In response, national guidelines in Sweden recommend family support programs to help manage unhealthy diet and physical inactivity in children [3]. Schools are widely recognized as ideal settings for delivering such health promotion programs, particularly for children aged 6–12 years [4, 5]. Implementing health promotion strategies in the schools context can be challenging for a number of reasons such as lack of cooperation between the home and the school [6]. All public schools in Sweden are governed at the municipality level and all schools are obliged to follow the Education Act [7] which states that school health care should work with health promotion and that health visits to the school nurse should occur regularly during primary and secondary education [7]. National guidelines specify that these visits should focus on promoting healthy behaviours such as physical activity, food consumption, and sleep in addition to regular health examinations and vaccinations [8]. Younger school-aged children rely on their immediate family for structure and support in developing healthy behaviours [9]. Additionally, parents might need support in creating a health-promoting environment for their school-aged children [10].

###  The healthy school start plus program

The *Healthy School Start Plus* [HSSP] is a universal four-component evidence-based program that targets parents and children during the first school year to promote healthy dietary and physical activity behaviours and to prevent or reduce child overweight and obesity. The program is delivered by teachers and the school nurse. The program has been evaluated in two previous trials Healthy School Start-1 [11] and Healthy School Start-2 [12] with three intervention components: (1) A health information brochure for parents available in five different languages and mainly focused on healthy behaviours for school-aged children; (2) A series of nine classroom lectures delivered by teachers to children with home assignments to be completed by the children together with their parents; and (3) Person-centred Motivational Interviewing (MI) [13] for parents delivered by an external expert focusing on healthy behaviours for children without the child’s presence [11, 12]. In the third trial, HSSP [14] the program had been adapted based on findings in previous trials. For example, the health brochure was adapted to include more concrete examples of positive parenting practices for promoting healthy behaviours in school-aged children. Also, the MI was delivered by the local school nurse instead of an MI expert as in study 1 and 2. The MI was offered as a complement to the regular health visit during the first school year and focused on how parents can promote child health via dietary, physical activity and sleep behaviours. School nurses had received 2½-day’s training in MI before the start of the intervention. School nurses were advised to focus the conversations on positive parenting practices related to the area of interest of the parent. The MI sessions were held at the school nurse’s office at the child’s school, and parents had received the printed health brochure prior to the MI. Finally, a fourth component was added to the program in this third trial, consisting of a validated online self-test for parents’ risk of type 2 diabetes (FINDRISC) [15] with direct automated feedback. The trial was registered on January 4, 2018, and made available online at ClinicalTrials.gov (ID: NCT03390725).

### Rationale

Schools are important platforms for the universal promotion of children’s healthy behaviours with the possibility to even reach parents. In Sweden, school nurses are key actors in disseminating and advocating health-promotion and disease prevention actions aimed at school-aged children guided by national guidelines. Therefore, understanding the experiences and perceptions of school nurses in delivering the HSSP program could be crucial for designing and implementing feasible and sustainable school-based health-promotion interventions in the future.

### Aim

The aim of this study was to explore school nurses’ perceptions and experiences of delivering a health-promotion program focusing on healthy lifestyle habits in primary schools in Sweden.

## Methods

### Study design

In this study, an explorative qualitative design with an inductive approach was used [16]. Qualitative methods are useful for exploring participants’ perceptions and experiences. The reporting checklists Consolidated framework for Reporting Qualitative research (COREQ) [17] was followed.

### Data collection

#### Setting

This study was part of the process evaluation of the HSSP, carried out as a cluster-randomised controlled trial [14, 18] in 15 primary schools (7 intervention schools) situated in disadvantaged areas (based on the average educational level of parents) in and around Stockholm, Sweden, during one school year (October 2017 to May 2018).

#### Participants

School nurses in all intervention schools in the HSSP trial were asked to participate in individual interviews focusing on experiences of working with the HSSP program. Participating school nurses (*n* = 7) were all women and, on average, 47 years old and had worked for 3 years as a school nurse.

#### Data collection

All participating school nurses were interviewed face-to-face at a location suitable for the interviewee or at the specific request of the school nurse by telephone (*n =* 1) after the end of the program. An experienced qualitative researcher (SA) conducted the interviews using a semi-structured interview guide (Supplement 1) based on barriers identified using the Consolidated Framework for Implementation Research [CFIR] [19, 20]. The interview guide was developed specifically for this study i.e., with the purpose to evaluate school nurses’ experiences of delivering the HSSP program. All interviews were audio-recorded and transcribed verbatim by a transcription service. A small part of the transcribed material was used in a previously published mixed-methods study, investigating school nurses’ experiences of using Motivational Interviewing as a conversational method [21]. Examples of questions that were posed during the interviews were: “*How did your school choose to participate in the HSSP program?*”, “*How did working with the HSSP program align with your ordinary work and responsibilities?”* and *“If you were to give some advice to school nurses just starting up the HSSP program*,* what would that be?”*

### Analysis

The data analysis followed the six phases of reflexive thematic analysis described by Braun & Clarke [22, 23] and was supported by QSR NVivo Version 12 [24]. The analysis was carried out by two of the authors (MM, SA) and involved an iterative movement between the 6 phases of analysis. First, all transcripts were read through several times to familiarise with the data, and an initial understanding of the content was noted. Next, data were coded through systematically searching and labelling phrasings corresponding to the studied phenomenon. Subsequently, all identified codes were sorted into initial themes that were created by the researchers making assumptions about the patterns that where found. In coherence with reflexive thematic analysis [23], the researchers’ prior knowledge of e.g., process evaluation and the CFIR framework [19, 20] most likely influenced phrasings of codes and initial themes. For example, the terms relevance, feasibility, acceptability, and adoptability, are commonly used in implementation science, and were also highly present in coding of the data. When all codes had been sorted into themes through the analysis software, all the initial themes were mapped and reviewed. Codes in each of the themes were revisited to ensure a coherent pattern and meaning. Codes found to belong in another theme were reorganised, and codes within a theme were further scrutinised to reflect the essence of data accurately. Example of the analysis process can be found in Table 1.

**Table 1:** Example of process in reflexive thematic analysis

Data	Codes	Initial themes	Final themes
*There should be some common guidelines. You [as a school nurse] usually work at a school alone*,* it becomes very much ‘I’ll do it my way’*,* which means that it will not be equal school health services. It [school health services] looks very different from school to school depending on how the municipalities choose to allocate their resources and money*	School nurse profession; Missing common guidelines; unequal services; political priority matters	Common and evidence-based procedures that bring health equality	Initial thoughts of the HSSP program
*[The intervention] maybe generated 20 more minutes or whatever it might have been*,* and I think that I would have had to spend those 20 min on that family another time anyway. So*,* on the whole*,* it doesn’t make any difference*,* it worked well. Before you think that ‘oh*,* this is a lot of time’ and ‘how am I going to make time for this’. But you have time*,* that’s what’s so strange. The more you do*,* the more time you have*,* really weird equation*	Time allocation; comparing procedures; relevance; acceptability; priority; feasibility	Relevance and feasibility of the program	Integrating the HSSP program into routine practices
*We should always think about health promotion*,* that’s kind of the central thing*,* and then I think that this is a very good way to go. I think it’s so good because it’s a collaboration between teachers and us*,* that it becomes much broader*,* than just us working with it. It comes into so many parts*,* also in the teaching. I wish that we can continue to be linked to something that the teacher also talks about in the classroom*,* so that it always becomes a collaboration*,* we did not have this quite the same way before*	Unexpected benefits; improved relations; engagement; Potential effects	Expected and unexpected effects of the program	Impact of the HSSP program

Next, naming final themes was an organic process, with the authors working together, reviewing, and revising initial formulations throughout the process. To ensure divergence, each theme was briefly described using illustrative descriptions and quotes to identify its essence. Finalised themes were presented with references to the original data for quotes to illustrate the essence of the data and theme. Similarities and differences were discussed through constant peer debriefing. All co-authors read and approved the descriptions and phrasings of the final results [25, 26].

## Results

This thematic analysis revealed that school nurses delivering a school-based health promotion program had extensive insight regarding the acceptability and appropriateness of the program as well as reflections regarding implementation and perceived impact of the program (Table 2).

**Table 2:** Results presented as themes and sub-themes

Themes	Initial thoughts of the HSSP program	Integrating the HSSP program into routine practices	Impact of the HSSP program
**Sub-themes**	Health promotion in the school nursing context	Navigating decision-making pathways	School nurses’ perspectives on parental engagement
	Balancing expectations and resources	Challenges, adaptations and opportunities during implementation	Enhanced collaboration between school nurses and teachers
			Being alone in program delivery: a lack of sustained leadership

### Initial thoughts of the HSSP program

This theme describes school nurses’ perceptions of the potential of the HSSP program, describing the perceived fit and relevance of HSSP for the school setting and how they balanced expectations and resources.

#### Health promotion in the school nursing context

Working with health promotion was perceived as part of the school nurse’s profession. Many of the school nurses referred to national guidelines where these expectations were expressed. Still, at the same time, the interviewees stressed that standardised structures and tools for equitably achieving these goals were not yet in place: “*There should be some common guidelines. You [as a school nurse] usually work at a school alone*,* it becomes very much ‘I’ll do it my way’*,* which means that it will not be equal school health services. It [school health services] looks very different from school to school depending on how the municipalities choose to allocate their resources and money*” (SN-6).

School nurses described their view on responsibility regarding children’s health as a balancing act. They explained that while parents have a significant responsibility for their children´s health, school nurses also have a responsibility to provide information and support for promoting healthy behaviours. As such, the school nurses discussed how the HSSP program could help address some of the known health disparities in modern society, such as inequalities related to unhealthy behaviours and unhealthy weight gain.

Many of the school nurses had heard of the conversational technique MI prior to the intervention and perceived the method as recommended by authorities as it is considered evidence-based and stressed that parents should be approached when promoting healthy behaviours to school-aged children: *The key [to children’s health] are the parents*,* so you won’t get anywhere if you don’t reach them. If I don’t reach the parents or manage to engage them*,* then it becomes pointless*” (SN-7).

Thus, the school nurses viewed health promotion as a core part of their professional role, yet they faced challenges due to a lack of common structures and the necessity of involving parents, which the HSSP program addressed.

#### Balancing expectations and resources

The HSSP program with health-promoting activities was perceived as in line with what school leaders and politicians expected from a school nurse practitioner. At the same time, the school nurses stressed the need for increased manpower to ensure equality in services provided and that these improvements boil down to political priorities: “*It would be absolutely fantastic if all schools were staffed up. I know that I am quite privileged*,* if you look nationally*,* there are those [school nurses] who have a lot more children than I do. Well*,* you can’t just count heads either*” (SN-1).

School nurses discussed that some tasks were more explicit in current legislation and steering documents, such as immunisations and regular health visits and were thus considered of higher priority for the allocated time: “*Time does not exist. That is what [the basic program] we have to do according to the National Board of Health and Welfare*,* otherwise I am committing misconduct*” (SN-2). This highlights how school nurses had to prioritise some tasks, often at the expense of broader health promotion activities, which illustrates the tension between expectations placed on them and the resources available.

### Integrating the HSSP program into regular activities

This theme describes school nurses’ perceptions of implementation success or failure of the HSSP regarding how they navigated through the decision-making pathways, the challenges and adaptations during program implementation.

#### Navigating through decision-making pathways

*Decision pathways regarding participation in the HSSP program varied between different school organisations. In some schools*,* the school nurses had received information about the HSSP program and argued to the principal for the school to participate. Other school leaders made the decision for the school to implement the program*,* without consulting the school nurses: “The decision [to participate in HSSP] was made up there [principal and central school health]. That they have a lot to do*,* I understand that*,* but that you [as a school nurse] was not even asked from the start*,* but rather ‘now we are going to do this’. I was bothered by that part. They just said yes without checking what it is or checking if everyone want to do it. We [school nurses] would probably have agreed anyway*,* so it’s not that*,* it’s more that you should maybe start talking to all the parties involved first before you say yes to something” (SN-3). This illustrates the difference in decision-making structures among schools*,* which influences how the program was implemented and perceived. Challenges*,* adaptations and opportunities during implementation*.

The MI was usually performed in connection with the regular health visit. In some schools, the regular health visit included a session with the school physician. Here the school nurse first performed height and weight measurement, and eyesight controls before the appointment with the physician. After this the school nurse held the MI. In other schools, the school nurse had full responsibility for all parts of the health visits and could integrate the MI more freely. Some MI were scheduled on a different time, suitable for the parent schedule.

Some school nurses perceived the HSSP as a somewhat awkward repetition of the regular health visit rather than an extension focusing on parenting issues. Also, adding the HSSP to the routine program was perceived as more time-consuming as compared with the standard health visit. Furthermore, some school nurses found it challenging and time-consuming to remind parents who often rescheduled the meetings due to work commitments or who had to bring younger siblings to the meeting. Nonetheless, despite this extra time required, it was considered valuable and a worthwhile investment: “*[The intervention] maybe generated 20 more minutes or whatever it might have been*,* and I think that I would have had to spend those 20 minutes on that family another time anyway. So*,* on the whole*,* it doesn’t make any difference*,* it worked well. Before you think that ‘oh*,* this is a lot of time’ and ‘how am I going to make time for this’. But you have time*,* that’s what’s so strange. The more you do*,* the more time you have*,* really weird equation*” (SN-1). Thus, although the HSSP program was initially perceived as demanding in terms of time, many school nurses came to view the additional effort as both feasible and worthwhile, since it addressed needs that would otherwise have required attention at a later stage.

Language and other cultural differences were commonly perceived challenges when meeting parents of different nationality. School nurses found that some parents with a non-Swedish background lacked common knowledge regarding healthy behaviours and that information regarding, e.g., healthy growth development and healthy food intake needed to be at a more basic level. Furthermore, it was perceived with scepticism to work with MI through an interpreter as it was uncertain what was translated and how it was said: “*MI was really difficult [via interpreter]*,* so I couldn’t have that. I know that I tried at some point*,* but I then understood that the interpreter had*,* based on the reaction from the parents*,* that it had gone wrong so that I kind of had to give it up a little bit*” (SN-4).

Some of the school nurses expected to continue implementing parts of the HSSP program, such as incorporating MI skills in conversations with older students and using classroom activities with teachers.

### Impact of the HSSP program

This theme outlines school nurses’ views on the reach and impact of the HSSP on three key groups: target group of the program (parents and children), deliverers of the different program components (school nurses, parents, and teachers), and decision-makers responsible for initiating the program (school health teams, principals, and municipal leaders).

#### School nurses’ perspectives on parental engagement

Parents told the school nurses what their children had learnt during classroom activities and their involvement in homework assignments. The school nurses also observed the positive impact of the HSSP program on the children during the regular health visits: “*They [the children] thought it was fun*,* and they started talking about this with Keyhole-labelled foods [officially health-branded] when they had talked about it in class. So*,* from the child’s perspective*,* I think it was only positive*” (SN-4). This way, the HSSP program could enhance children’s health literacy in ways that were visible both for the school nurses at school and in conversations with parents.

School nurses recognised that parents with greater support needs might be more challenging to engage. According to the school nurses, some parents were actively engaged in the HSSP program, while others were not: “*They [the parents] wanted their children to be healthy and things like that*,* but that they [the parents] would do something themselves wasn’t quite as sure*” (SN-5). This reflected how parental engagement varied from active collaboration to more passive expectations, and the need for strategies to reach less motivated parents.

The school nurses mentioned that parents showed appreciation during health conversations, such as through smiles and verbal gratitude, or later, with cheerful greetings in the school corridors, providing updates on their progress or asking for more guidance: “*I’ve noticed*,* that the parents I met at a Health school start*,* they still [later grades] like to come to me with their questions. You got a special relationship when you sat and talked*,* and that the health conversation became very different than it was before. I think those parents felt listened to and you got a different relationship with those parents*” (SN-4). In this way, the program was seen as strengthening the relational dimension of school health care, where parents felt heard and were more likely to continue seeking guidance beyond the intervention itself.

#### Enhanced collaboration between school nurses and teachers

School nurses described increased cooperation between school nurses and teachers during the program, increasing the understanding of the other´s professional duties. Teachers, who meet the parents naturally during the school day, helped remind them about their scheduled health conversations. At some schools, the school nurses occasionally supported teachers during classroom activities on their own initiative. At other schools, nurses advocated that more guidance regarding possible synergies would have been beneficial: “*As a school nurse*,* I think it would have been fun to be part of the lessons. Now I could read up on what they were going to do*,* but it would probably have been fun to go in and have some education myself for the children”.* (SN-4)

School nurses described that some teachers were not used to addressing HSSP topics in the classroom. According to the school nurses, the teachers took more responsibility for health promotion through their participation in the program. This approach was considered a valuable opportunity for collaboration: “*We should always think about health promotion*,* that’s kind of the central thing*,* and then I think that this is a very good way to go. I think it’s so good because it’s a collaboration between teachers and us*,* that it becomes much broader*,* than just us working with it. It comes into so many parts*,* also in the teaching. I wish that we can continue to be linked to something that the teacher also talks about in the classroom*,* so that it always becomes a collaboration*,* we did not have this quite the same way before*” (SN-4). The HSSP programme was judged to be a good way towards shared responsibility for health promotion, between the teachers and school nurses.

#### Being alone in program delivery: a lack of sustained leadership

It was a common perception that although the principal had been involved at the initiation of the project, the school nurses and teachers had been left to manage the HSSP program on their own: “*It was just like ‘yes*,* of course we’re going to be involved’ and then it was over for her part [the principal’s]. She [the principal] was in charge but never asked me*,* ‘how are you*,* what do you think’*”. (SN-3)

At the same time, interviewees stressed that schools are often overwhelmed with school-based projects and interventions of different sorts and that these usually come and go without further sustainment. The school nurses emphasised that strong and consistent support from school leadership are crucial to ensure that programs like HSSP become sustainable.

## Discussion

In this study, we aimed to explore school nurses’ perceptions and experiences of delivering a health-promotion program together with teachers focusing on healthy lifestyle habits in Swedish primary schools. The results revealed that school nurses appreciated the evidence-based content and structure of the HSSP program. However, implementation challenges arose due to increased workloads and declining interest from school principals. Additionally, difficulties in reaching vulnerable families with language barriers were noted. Yet, examples of parental appreciation highlighted the importance of personalised interactions in health promotion.

School nurses play a crucial role in promoting health and well-being among children, and they are expected to provide evidence-based care. The nurses expressed appreciation of the clarity and evidence-based nature of the HSSP materials. Previous research has highlighted that a well-structured health-promotion content and comprehensive educational resources positively impact school nurses’ confidence in health-promotion practices [27, 28]. Thus, the HSSP material provided a valuable contribution to the school nurses´ work with health promotion. The school nurses recognised that, by assisting school nurses with an evidence-based intervention, HSSP has the potential to address health disparities that are highly prevalent in modern society, particularly concerning unhealthy behaviours, overweight and obesity. Supporting this supposition, a pooled analysis of the three RCT trial of the HSSP program showed that it had significantly reduced the BMI z-score in children with obesity at baseline [29].

Our study underscores the challenges faced by school nurses due to competing and demanding activities, which can hinder sustained implementation of a program like the HSSP. Balancing health promotion alongside other compulsory health assessments posed a challenge. Previous studies have shown that school nurses are motivated and feel competent to work with health promotion, but are not always given the appropriate opportunities [30]. Previous research from Greece [31] has also highlighted common obstacles, including time constraints and competing interests for school nurses. By allocating more time for health promotion school nurses could be supported in their roles [32]. Additionally, Ryan [33] stressed the importance of active involvement by school nurses in developing health promotion programs. This aligns with the findings from England [34] in a qualitative study on school-based health promotion in practice, where school nurses and students suggested that health needs to be prioritised, with strong leadership support, a common goal and fruitful collaboration.

The decline in some school principals’ interest after initial enthusiasm, as highlighted in our study, has also been reported in a systematic review [35]. This review emphasises the critical role of school leaders in transitioning health promotion programs into actions and sustaining them over time. Furthermore, their study suggests that supportive leaders can enhance the implementation of health promotion initiatives, while weak support can hinder implementation. From this perspective, sustained health promotion in schools may require, as also suggested by nurses in our study, a “whole-school approach”, including facilitators with a role to continuously engage staff, from the school principal, school health team, teachers, students to parents. The increased collaboration between school nurses and teachers is a promising step towards better collaboration and a more comprehensive health promotion approach in schools.

In our study, school nurses emphasised the importance of personalised interactions with the parents during health conversations. Previous studies have also highlighted the positive outcomes associated with these interactions, underscoring the need for parental involvement in health promotion efforts [36, 37]. Parents perceive conversations about children’s health with the school nurse as valuable support and motivation to promote children’s healthy behaviours [21]. However, the school nurses also stressed the challenges of language barriers and complex family needs in their respective schools, which hindered communication with parents. Wahlström et al. [38] have pointed out the importance of culturally sensitive approaches in engaging diverse families. In the updated version of the HSS program, the parental health brochure (now called “parental guide”) is available in 11 of the most common languages spoken in Sweden. Furthermore, Ellertson et al. [39] demonstrated that nurses perceive physical and mental health to be worse in children from disadvantaged areas than in areas with a population with higher socioeconomic status. This perception is also corroborated in public health reports [2]. These high-risk areas typically include communities with a large proportion of inhabitants with a non-Swedish background who receive financial aid. Thus, finding a way for school nurses to effectively engage with families facing language barriers and other needs is essential in health promotion efforts in the school context with the aim of reducing social inequalities in health. Here, systematic health promotion programs, emphasising a multifaceted whole-school approach, should be beneficial to reach and engage even high-risk families.

To summarise, the above discussion indicates the following implications:

- Enhance collaboration between groups of staff, e.g., teachers and school nurses, to facilitate work on health promotion in schools.
- Allow more time for school nurses to work with health promotion.
- Include school nurses in the development of future health school-based promotion intervention.
- Provide additional facilitating material, support, and training for school nurses in how to communicate with parents who have a low proficiency in Swedish.

The study sample and setting indicate some limitations to the study. All school nurses were women and only seven schools were represented in the study. This indicates that transferability must be interpreted with caution. It is likely that school nurses in other geographical areas may perceive working with the HSSP differently. However, schools were situated in areas considered to be disadvantaged. This can be considered a strength of the study since it includes perspectives of relevance to addressing the socioeconomic gradient in overweight and obesity. An additional strength is the use of implementation theory in the study, where CFIR was used to inform the development of the interview guide. This use of theory mitigated the risk of overlooking aspects of importance for implementation that have previously been found in the literature.

Also, the data were collected between 2017 and 2018, which may raise concerns about their current relevance. Nonetheless, the core challenges and facilitators identified—such as time constraints, collaboration with teachers, and strategies for reaching families—remain highly pertinent in today’s school health context. The intervention studied is still in use and continues to inform practice, supporting the ongoing relevance of the findings.

Trustworthiness was of the study was strengthened by the use of illustrative quotes, and the audit trail described in the methods section. In addition, the analysis was conducted by researchers with different backgrounds and perspectives, but all with extensive experience from conducting qualitative studies.

## Conclusion

This study sheds light on the complexities faced by school nurses in delivering the MI component within a health promotion program in primary school together with teachers. Our findings underscore the critical role that school nurses have as advocates, educators, and facilitators for school-based health promotion to reach families and the home environment. While evidence-based content and structured approaches are valued, challenges such as extra time and competence needs, declining leadership support, and communication barriers can hinder successful implementation. Personalised interactions remain a cornerstone of effective health promotion practices, emphasising the need for tailored approaches to engage both school children and their parents. Moving forward, evidence-based practices, sustained leadership support, and culturally sensitive strategies are essential for successful health promotion within schools.

## Supplementary Information

Below is the link to the electronic supplementary material.

Supplementary Material 1

## Data Availability

The data that support the findings of this are available from the corresponding author upon reasonable request. Data are located in controlled access data storage at Karolinska Institutet.
