# Supplementary material for: School nurses’ perceptions and experiences of delivering a universal health-promotion program targeting both children and parents in the Swedish primary school context
Source: BMC Nurs. 2025 Sep 2;24:1158. doi: 10.1186/s12912-025-03806-2 (PMC12406544; doi:10.1186/s12912-025-03806-2)
Supplement: Supplementary file 1 — Supplementary Material 1 [file 12912_2025_3806_MOESM1_ESM.docx]

**Interview guide for nurses in HSSP**

**Introduction**

*Introduction to the purpose of the interview. To provide space for reflection, make use of experiences, understand how the program is experienced and how it is used.*

- Tell us how it came about that you (in your school) joined this project?
- Can you tell us how you first came into contact with HSSP?
- How do you perceive that HSSP worked over all?
- What worked better/less well?

**Topic: Content of the HSSP (bring material)**

*Introduction: a large part of the project for you as a school nurse has been to have Motivational Interviewing (MI) conversations with parents*

- Can you tell us about your work with these conversations?
- How have the conversations worked? (what made it work better and worse?)
- How did you feel that the Motivational Interviewing method worked?
- What was easier/harder to work with? What was missing?
- How did you experience the work?
- Considering your background as a nurse and the prior knowledge you have, how easy or difficult did you find it to carry out the MI conversations? (sufficient prior knowledge?) (CoI)
- How did you experience the education in MI? (level, knowledge, time consumption, possible improvements to the education)
- How did it work to work with HSSP plus in combination (together) with your regular work at school? (IC)
- What would need to be adapted (changed) to make it better/easier to work with the project? (IC)
- Did you make any adjustments to your way of working?
- What support do you think you need as a school nurse to implement HSSP plus?
- How did the parents receive the conversations?
- How do you feel that the MI conversations contributed to changing the children's habits?

**Topic: Relationship with parents**

*Introduction: Now I have some questions about the role of parents.*

- Can you tell us about your impression of the parents' work with HSSP plus? (Parent ability)
- When it comes to a project like this, that aims to promote healthy eating and movement habits and prevent obesity in children, what responsibility do you think you have as a school nurse compared to the children's parents?

**Theme: School**

*Introduction: Leaving practical work with the project and focus on the implementation at your school.*

- How did you experience the decision to have the project at your school?
- How was it decided that you would join HSSP? (How did you experience that process).
- During the implementation of the project, who would you say has been the main responsible for HSSP at the school?
- Who (more people) have been involved in HSSP at the school?
- How has your collaboration with involved colleagues been (those mentioned before)?
- Can you give examples of whether and if so, how you have collaborated with teachers and other staff?
- How do you feel that HSSP has been received by other staff at the school?
- Has the work with HSSP affected anything else in the school, if so, how?
- In what way has the school's management been involved in the implementation of ETUC? (How do you experience that? What would you need?)
- What capacity/conditions were there at your school to work with HSSP plus? (IS)

**Theme: Communication and information**

*Introduction: If we look at this with communication and information in connection with HSSP*

- What has the communication been like about the project?
  (With others at the school, school management, parents, Karolinska Institutet)
- How do you feel that the information about HSSP has been?

**Theme: Role of the school nurse**

*Introduction: It would also be good to hear your reflections on the importance of the project for school health care and if the HSSP method were to be implemented in other school.*

- How would it fit into the school nurse's regular work?
- How does the working method within and area of the project correspond to current policy documents for you as a school nurse? (national guidance, job description, etc.)
- Have you continued to work with the working method in HSSP after the end of the project?
- What would be needed for you as a school nurse to continue working with this?
- What structures or political decisions would be relevant for working with these issues in school health care?

**Conclusion**

- What would you have liked to do differently if you redesigned HSSP?
- If you were to give any advice to a school nurse who is just starting with HSSP, what advice would you give?
- Now I have no questions left, except for one last one, what have I missed to ask about?

**Facilitating questions:**

- what influenced,
- how could it be better,
- what would be needed,
- give examples,
- describe how you mean
